# Attention and working memory in patients with prolactinomas: a case–control study

**DOI:** 10.1038/s41598-022-26331-7

**Published:** 2022-12-29

**Authors:** Aleksandra Bala, Tomasz Dziedzic, Agnieszka Olejnik, Andrzej Marchel

**Affiliations:** 1 Faculty of Psychology, University of Warsaw, Stawki 5/7, 00-183 Warsaw, Poland; 2 Department of Neurosurgery, Medical University of Warsaw, Żwirki i Wigury 61, 02-091 Warsaw, Poland

**Keywords:** Pituitary diseases, Psychology, Pituitary diseases

## Abstract

Prolactinomas (prolactin-secreting adenomas) are the most common type of hormone-secreting pituitary tumor. Mounting evidence indicates that excess prolactin impairs cognitive function, but specific assessments of attention in patients with prolactinomas are lacking. Case–control study gathered 54 participants—27 patients with prolactinoma and 27 healthy controls. Neuropsychological assessment included a comprehensive set of diagnostic methods for the evaluation of attention and working memory. Patients showed slower information processing, expressed as a longer working time on the d2 Test of Attention and Color Trails Test (CTT-2), and lower attention-switching shown in the CTT-2 and in two subtests of the Tests of Everyday Attention (Visual Elevator), and Telephone Search While Counting. Working memory disturbances were observed in Digit Span and Symbol Span tests. A level of prolactin correlated negatively with scores in some of the neuropsychological tests measuring attentional switching (Visual Elevator), spatial screening and working memory (CTT-2), spatial working memory (Symbol Span) and auditory-verbal working memory (Digit Span backwards). There were no significant correlations between cognitive performance and tumor size. In conclusion, patients with prolactinoma suffer from impaired cognitive functions, including attention and working memory. Comprehensive neuropsychological assessment should be a permanent element of the diagnostics of this group of patients.

## Introduction

Pituitary tumors (adenomas) are common, occurring in approximately 10–15% of the general population worldwide^1^. The most frequently occurring pituitary tumors are prolactinomas, which account for up to 45% of pituitary adenomas. They elevate blood levels of prolactin and affect the body’s tissues and function, leading to hypogonadism and/or galactorrhea in both sexes, decreased libido and sexual potency in men, and amenorrhea and infertility in women^2^.

Following several case reports of cognitive impairment due to hyperprolactinemia^3–5^, some group studies confirmed this relationship. In one group of men, higher prolactin levels correlated negatively with cognitive function and well-being scores, and positively with scores in depression scales^6^. In another study, group of men with giant prolactinomas presented with cognitive decline: frontal syndrome, and memory problems, as well as unconsciousness^7^. Furthermore, men and women with prolactinomas had significantly lower scores on verbal and non-verbal memory and attention than healthy volunteers^8^. Another study showed, that when cabergoline was used to lower prolactin in patients with hyperprolactinemia, this improved cognitive function^9^. Cognitive and neuroimaging studies by Yao and colleagues found a relationship between hyperprolactinemia, grey matter loss and cognitive dysfunction in women with prolactin-secreting tumors^10^, and altered thalamocortical and cerebellar-cerebral functional connectivity (FC)^11^ in men and women with prolactinomas. Frontal theta oscillation, the electrophysiological marker of executive dysfunction, was also identified in patients with prolactinomas^12^. Another electrophysiological study further suggested that treatment of prolactinomas may improve deficits in response activation and inhibition at the cognitive and electrophysiological level^13^.

In our clinical experience, attentional problems are among the most common complaints in patients with prolactinomas, yet despite the considerable advances in the understanding of cognitive processes in patients with prolactinomas, there has been no comprehensive evaluation of attention in this group of patients to date. As is well known, attention is a very complex group of cognitive processes with a complex, hierarchical structure^14^, which means that its fragmentary assessment does not fully reflect the clinical picture and does not allow the full understanding of the difficulties that the patient is struggling. The aim of the present study was a comprehensive assessment of the attention processes and working memory in patients with prolactinomas. We hypothesized that patients would score lower than healthy participants in measures of attention and the level of attention disorders will be associated with the level of prolactin overproduction.

## Results

The patient and control groups did not differ in any demographic characteristics (Table 1) but there were significant between-group differences in most of the neuropsychological test scores (Table 2).

**Table 1:** Sociodemographic and clinical characteristics of patients with prolactinoma and healthy controls.

	Prolactinoma (*n* = 27)	Controls (*n* = 27)	Two tailed *t* test or Chi-square test
Age, M ± SD	35.19 ± 15.22	34.89 ± 16.01	t_df52_ = 0.19, *p* = 0.84
Sex (F/M)	15/12	15/12	
Handedness (R/L)	25/2	26/1	χ^2^_df1_ = 1.11, *p* = 0.45
Years of education, M ± SD	15.65 ± 2.36	15.04 ± 2.71	t_df52_ = 0.41, *p* = 0.53
Prolactin level, M ± SD	Whole group: 291.12 ± 107.45	Whole group: 17.33 ± 5.29	Whole group: t_df52_ = 0.09, *p* < 0.001
	F: 295.14 ± 97.63^a^	F: 18.12 ± 4.91^a^	**F: t**_**df28**_** = 0.11, *p* < 0.001**
	M: 281.10 ± 112.57^b^	M: 13.06 ± 3.62^b^	**M: t**_**df22**_** = 0.08, *p* < 0.001**
Tumor volume in mm^3^, M ± SD	224.38 ± 141.78	N/A	

Significant scores are shown in bold.

*M* mean, *SD* standard deviation, *F* female, *M* male, *R* right, *L* left, *N/A* not applicable, *ng/mL* nanogram/milliliter.

Normative values for prolactin—^a^F: 4.79–23.3 ng/mL; ^b^M: 4.04–15.2 ng/mL.

**Table 2:** Test results of patients with prolactinoma and healthy controls.

		Prolactinoma M ± SD	Controls M ± SD	Two tailed *t* test
CTT	CTT-1 times	43.21 ± 10.25	38.13 ± 9.12	t_52_ = 0.39; *p* = 0.092
	CTT-1 errors	0.63 ± 0.49	0.56 ± 0.37	t_52_ = 0.43; *p* = 0.110
	**CTT-2 times**	**113.41 ± 12.37**	**62.85 ± 11.64**	**t**_**52**_** = 6.33; *p* = 0.001**
	**CTT-2 errors**	**2.11 ± 1.99**	**0.81 ± 0.42**	**t**_**52**_** = 1.09; *p* = 0.012**
	**Interference score**	**1.66 ± 1.12**	**1.18 ± 0.31**	**t**_**52**_** = 1.48; *p* = 0.016**
**Symbol span**		**14.95 ± 4.11**	**26.16 ± 3.28**	**t**_**52**_** = − 3.76; *p* < 0.001**
D2	**Number of processed items**	**270.55 ± 168.13**	**405.35 ± 191.18**	**t**_**52**_** = − 1.06; *p* = 0.023**
	Sum of errors	36.33 ± 21.06	31.49 ± 19.74	t_52_ = 0.54; *p* = 0.423
**Visual elevator**	**Number of correct**	**5.84 ± 2.13**	**8.77 ± 1.09**	**t**_**52**_** = − 2.58; *p* = 0.035**
	**Time score**	**4.81 ± 2.17**	**3.49 ± 1.01**	**t**_**52**_** = 1.07; *p* = 0.006**
Elevator counting		6.51 ± 0.38	6.89 ± 0.09	t_52_ = − 0.51; *p* = 0.812
**Digit span**	Forward	5.64 ± 2.11	7.23 ± 2.61	t_52_ = − 2.33; *p* = 0.051
	**Backward**	**4.73 ± 2.98**	**7.21 ± 2.78**	**t**_**52**_** = − 2.16; *p* = 0.015**
Telephone search		2.93 ± 0.38	2.61 ± 0.41	t_52_ = 0.79; *p* = 0.871
**Telephone search while counting**		**3.92 ± 1.75**	**0.62 ± 0.43**	**t**_**52**_** = 3.26; *p* = 0.009**

Significant scores are shown in bold.

*M* mean, *SD* standard deviation, *s* seconds, *CTT* color trails test, *D2* D2 test of attention.

### Patients with prolactinomas show impairments in processing speed and working memory

The patient group had lower scores than the control group in the D2 test, which measures selective attention. Although the number of errors did not differ between the groups, the processing speed (measured by the number of characters viewed) was significantly slower in the patient group.

Similarly, in the CTT—a test of spatial screening and working memory—patient group took significantly longer than the control group to complete the second part of the test, the number of errors was also higher. There were no differences in the number of errors nor time taken to perform the first part. Moreover, the interference score was significantly higher (worse) in the patient group.

Scores in the Digit Span forward and backward tasks as well as the Symbol Span tasks were significantly lower in the patient group than in the control group.

### Patients with prolactinomas show impairments in attentional switching but not sustained attention

There were no differences in sustained attention measured using the Elevator Counting subtest of the TEA.

In attentional switching measured with the Visual Elevator subtest of the TEA, the patient group scored significantly lower in both accuracy (number of correct answers) and speed (average time taken to perform correctly solved tasks).

Patients also achieved lower results than controls in the Telephone Search subtest of the TEA, but this was only on the level of trend (not statistically significant). In the second part of this task (Telephone Search While Counting), patients achieved significantly lower scores than controls.

### Severity of cognitive impairment in patients with prolactinoma is related to the prolactin level but not tumor size

Correlation analyses showed that higher prolactin levels were associated with lower scores in some working memory and attention assessments, namely attentional switching (Visual Elevator: accuracy, *r* = − 0.52, *p* < 0.05; timing, *r* = − 0.42, *p* < 0.05), spatial screening and working memory (CTT2: speed of performance, *r* = 0.39, *p* < 0.01), spatial working memory (Symbol Span: *r* = − 0.49, *p* < 0.001) and auditory-verbal working memory (Digit Span—backwards: *r* = − 0.57, *p* < 0.01). There was no association between test results and the level of prolactin in healthy people, which means that fluctuations in the level of this hormone within the normal range do not have a significant effect on functioning. There were no significant correlations between cognitive processes and tumor size.

### Hyperprolactinemia affects the cognitive functions of women and men differently

Additional correlation analyzes were carried out between the level of prolactin and cognitive functioning separately for women and men. In the group of women, significant correlations were found in attentional switching (Visual Elevator: accuracy, *rho* = − 0.58, *p* < 0.01; timing, *rho* = − 0.61, *p* < 0.01), spatial screening and working memory (CTT1: speed of performance, *rho* = 0.32, *p* < 0.05; CTT2: speed of performance, *rho* = 0.42, *p* < 0.01), spatial working memory (Symbol Span: *rho* = − 0.55, *p* < 0.01), auditory-verbal working memory (Digit Span—backwards: *rho* = − 0.4, *p* < 0.05), as well as selective attention and concentration (D2: number of screened characters, *rho* = 0.37, *p* < 0.05). In men significant correlations were found in spatial screening and working memory (CTT2: speed of performance, *r* = 0.39, *p* < 0.01), spatial working memory (Symbol Span: *r* = − 0.49, *p* < 0.001), auditory-verbal working memory (Digit Span—backwards: *rho* = − 0.61, *p* < 0.01) and sustained attention (Elevator Counting, *rho* = 0.47, *p* < 0.05).

## Discussion

In the present study, patients with prolactinomas scored significantly lower than participants in the control group in tests of selective attention, attentional switching, divided attention, spatial screening and working memory (verbal and visual). Some aspects of attention remained at a level similar to healthy people. It is due to the fact that individual aspects of attention are represented by separate brain networks^15^ so it is possible that some of them have suffered from hyperprolactinemia more than others. More accurately describing this phenomenon certainly requires further research.Although the literature on the relationship between hyperprolactinemia and attention is sparse and in most of the previous studies, not the entire attention profile was assessed, but its selected aspects, our results are in concordance with previous findings^8^. To our knowledge, no research to date has focused directly on attention in patients with prolactinomas, but there are data from studies of patients with psychosis in whom hyperprolactinemia was inversely correlated with processing speed^16,17^, indicating that prolactin damages this aspect of attention. In addition, some studies of executive function^10,12,13^ also examined aspects of attention.

Hyperprolactinemia in patients with prolactinomas may affect brain function directly or indirectly^18^. The direct effect involves prolactin receptors that are widely distributed in the neural tissue, meaning that prolactin overproduction can affect neurotransmission, functional circuits, and neuronal signaling pathways across the entire brain^19^. The indirect effect results from an imbalance of other hormones and neurotransmitters. For example, prolactin inhibits testosterone and estrogen, which also play a role in axonal guidance, synaptogenesis and neuronal morphology^20^.

In the healthy population, prolactin regulates its own release through short-loop feedback. Prolactin receptors are present in the dopaminergic neurons of the arcuate and periventricular nuclei, where they influence the tuberoinfundibular dopaminergic pathway, one of the four major dopamine pathways in the central nervous system. Higher levels of prolactin cause an increase in the production of dopamine, which in turn inhibits prolactin, resulting in homeostasis^21^. An imbalance between prolactin and dopamine can lead to cognitive disorders^22^. The right level of dopamine is crucial for the functioning of the prefrontal cortex, and its disruption affects cognitive processes involving this brain region^23^, such as executive function, working memory, and attention^24–26^. Highly elevated levels of dopamine impair various cognitive domains, mostly those related to the prefrontal cortex and subcortical regions^27,28^. It can be assumed that the attention deficits seen in our study might be, inter alia, due to a disruption of the prolactin–dopamine homeostasis.

Prolactin distribution and expression influences brain morphology and function^29^. Hyperprolactinemia due to pituitary tumors can cause a decrease in gray matter volume in the whole prefrontal cortex^10^, which may explain the cognitive impairment (including attention deficits) observed in our patient group, but data on this are scarce. Some studies in patients with pituitary tumors have identified altered frontal theta oscillation, which can be a marker of executive dysfunction^12^, and altered thalamocortical and cerebellar-cerebral functional connectivity as well as an association between functional connectivity and prolactin levels^11^.

In the present study, we showed that the level of overproduction of prolactin in patients with prolactinoma correlated with the level of cognitive functioning, which is in line with the results of other studies^8,10^. The level of overproduction of prolactin usually depends on the tumor size ranging from below 200 ng/ml for tumors with a diameter less than 1 cm, 200 ng/ml to 1000 ng/ml in tumors 1 to 2 cm, and more than 1000 ng/ml in tumors sized more than 2 cm^30^. Since, as proven in this study, the level of prolactin correlates with the level of neuropsychological deficit, and the level of prolactin depends on the tumor size, one could expect that the tumor size will also correlate with the deficit level. However, this did not happen in our research. Explanation of this phenomenon may be that either some prolactinomas were not well-differentiated or there was a presence of cystic component in the tumor. In these cases, tumor size may not correspond with its secretory activity^30^ and this was probably the case in the patient’s group we examined.

Prolactin modulates the secretion of sex hormones, so one could expect that hyperprolactinemia will affect the functioning of representatives of both sexes in a different way. This study demonstrated that hyperprolactinemia affects the cognitive functions of women and men differently, but due to the small number of participants the results should be treated with caution and replicated in the future on a larger group. Prolactin may be also associated with the occurrence of psychopathological disorders, including depression, therefore another important research direction should include the analysis of this issue in the context of the observed cognitive disorders. Another limitation of this study is the lack of functional neuroimaging or electrophysiological methods, which would provide interesting information about the nature of attention disorders in prolactinoma patients. This is the direction of further research we are planning.

In conclusion, patients with prolactin-secreting adenomas have cognitive impairment, which has already been shown in previous research. Studying the specificity of these deficits and better understanding the problems faced by patients with prolactinomas is key to providing them with appropriate medical and neuropsychological care, and thus increasing their quality of life.

## Methods

### Participants

We recruited 54 participants (27 patients with prolactinoma and 27 healthy controls) for this case–control study. The patient group was recruited from inpatients admitted to the Medical University of Warsaw’s Department of Neurosurgery for assessment and treatment of prolactinoma. All patients were assessed by neuropsychologist before introducing pharmacotherapy. We excluded subjects with a history of neurologic or psychiatric disorders, those who were taking medication (including cabergoline, bromocriptine and oral contraceptives), were pregnant, had a history of drug or alcohol abuse, or had significant visual field defects that could prevent them completing the neuropsychological tests. The control group comprised healthy volunteers who were not pregnant or taking any medication. Both groups were matched in terms of sex, age, education, handedness, and mood level (Table 1).

### Procedure

The participants were informed about the aim of the study and details of the neuropsychological examination, then we obtained their informed consent to participate. Next, we interviewed them in a standard manner to collect information about their demographics and health, and performed the neuropsychological assessment (described in subsection “Neuropsychological assessment”). Patients were evaluated within the first 3 days of hospitalization, before introducing any pharmacotherapy for their prolactinomas. Both groups were assessed under the same conditions. The participants were tested once. The examination was performed as instructed in the tests’ manuals and took approximately 45 min. None of the subjects needed to take a break in the middle. All procedures were in accordance with the Declaration of Helsinki and were approved by the Committee for Ethics of Research at the Faculty of Psychology, University of Warsaw.

### Neuropsychological assessment

The neuropsychological assessment was performed in a hospital room without distractions. We examined specific aspects of working memory and attention using the comprehensive set of neuropsychological tests described below.

#### Selective attention and concentration: D2 Test of Attention^31^

Participants were presented with a piece of paper on which there were 14 test lines each containing 47 characters: “d” and “p”, each marked with one, two, three, or four dashes above or below the character. They were then asked to cross out target characters (all instances of “d” with only two dashes). There was a time limit of 20 s for completing each row. We assessed the number of screened characters and number (proportion) of errors.

#### Spatial screening and working memory: Color Trails Test^32^

This test comprises two subtests. The first part (CTT1) requires participants to connect numbered circles in sequence (1–25). We assessed the time taken to complete the task and the number of errors (wrong number in sequence). In the second part (CTT2), participants were again required to connect numbered circles, but this time each number was present in two colors (yellow and pink) and the goal was to alternate between them. We assessed the time taken to complete the task, and the number of errors (wrong number and/or wrong color). We also calculated the interference score as follows: (times to complete CTT2 − times to complete CTT1)/times to complete CTT1.

#### Sustained attention: Elevator Counting (a subtest of the Test of Everyday Attention; TEA)^33^

We asked participants to imagine that they are travelling in an elevator and to count a series of recorded tones that symbolize successive floors passing. After an initial practice of two strings, the subjects were presented with seven strings of tones, each of them was presented only once. The time to answer was unlimited. We assessed the number of correct answers.

#### Attentional switching: Visual Elevator (a subtest of the TEA)^33^

Participants were asked to imagine travelling in an elevator and to count visually presented elevator doors (symbolizing floors). The doors were labelled with arrows indicating the direction of travel (up or down) and participants had to count up or down and say on which floor the elevator finally stopped. Each person performed ten tasks. The time to complete each task was not limited, but it was measured. We assessed the number of correct answers and time of performance.

#### Visual screening and divided attention: Telephone Search, and Telephone Search while Counting (subtests of the TEA)^33^

Participants looked for certain symbols while searching entries in a “telephone directory”. When they noticed two given symbols (e.g. squares) in a row, they had to circle them as quickly as possible with a pen. In the first part of the test, it was the participants’ only task. In the second part, participants were required to count a series of tones while performing their search. They were instructed to work as quickly and accurately as possible and to put maximum effort into both tasks (counting tones and searching for symbols). The number of tone counting items depended on the speed of searching the telephone directory. There were many series of tones (recorded one after another with a pause of several seconds in between), and the task ended when the subject finished his visual search of the book, even if the tone-string was on-going. We assessed the time taken to complete the task and the number of correct answers.

#### Auditory-verbal working memory: Digit Span (a subtest of the Wechsler Adult Intelligence Scale)^34^

We asked participants to repeat a sequence of numbers read out loud by the researcher. The numbers had to be repeated in the same order in the first part of the test, and backwards in the second part. The sequences were of increasing length, each time two items of the same length were presented, the next two items longer by one element, etc. The task ended after both sequences of numbers of the same length were repeated incorrectly. We assessed the number of correct answers.

#### Spatial working memory: Symbol Span (a subtest of the Wechsler Memory Scale)^35^

In this test, the researcher showed the participant a set of abstract symbols in a row (from one to seven items) for 5 s. Next, the participant was shown another set of symbols that included the previously seen items as well as distractors, and was asked to point out the previously seen items in the order in which they were first presented. The time to answer was unlimited. The task ended after four incorrect answers in a row. We assessed the number of correct answers.

### Medical data

Magnetic resonance imaging (MRI) data and blood test results confirming the presence of prolactinomas were all obtained from the patients’ medical records with their informed consent.

### Statistical analysis

Statistical analyses were performed using SPSS version 24.0 (IBM Corp., Armonk, NY) for Windows. The Shapiro Wilk test showed that our data follow a normal distribution, therefore the comparisons between two independent samples (controls vs. patients) were done by Student’s *t* test. Associations between tumor size as well as level of prolactin and neuropsychological tests scores were examined using Pearson correlations. Results were considered significant when p < 0.05 (2-tailed testing).

## Data Availability

The datasets analyzed during the current study are available from the corresponding author on reasonable request.
